# Adolescents’ beliefs and perceptions of acne vulgaris: A cross-sectional study in Montenegrin schoolchildren

**DOI:** 10.1371/journal.pone.0253421

**Published:** 2021-06-16

**Authors:** Milena Ražnatović Đurović, Janko Janković, Milica Đurović, Jelena Spirić, Slavenka Janković

**Affiliations:** 1 Clinic of Dermatology and Venereology, Clinical Center of Montenegro, Podgorica, Montenegro; 2 Faculty of Medicine, University of Montenegro, Podgorica, Montenegro; 3 Institute of Social Medicine, Faculty of Medicine, University of Belgrade, Belgrade, Serbia; 4 Faculty of Medicine, University of Belgrade, Belgrade, Serbia; 5 Institute of Epidemiology, Faculty of Medicine, University of Belgrade, Belgrade, Serbia; Clinic for Infectious and tropical diseases, Clinical centre of Serbia, SERBIA

## Abstract

**Background:**

Acne is a common chronic inflammatory skin disease with a high prevalence in adolescent and early adult years. The aim of this study was to assess the self-perceived beliefs of Montenegrin secondary school pupils regarding the acne aggravating and ameliorating factors.

**Methods:**

This cross-sectional survey of the pupils was conducted during October and November 2020 in four randomly selected public secondary schools in Podgorica, Montenegro. All 500 pupils were asked to fill in a questionnaire that included questions on age, sex, presence of acne, perceived acne aggravating and ameliorating factors, and sources of their information about acne.

**Results:**

A total of 500 pupils, 234 (46.8%) boys, and 266 (53.2%) girls, aged 14−17 years, participated in the study. Acne was self-reported in 249 (49.8%) pupils, whereas 251 (50.2%) did not report acne. Factors most often believed to aggravate acne were inadequate face washing (85.0%), hormones (84.0%), sweets (82.0%), greasy food (72.6%), makeup (71.2%), and stress (67.8%). Overall the most prevalent acne ameliorating factors were cosmetic treatment (80.4%), increased water consumption (77.6%), a diet change to a healthier food choice (77.4%), and being on school holidays (62.2%). Girls reported more frequently that genetics, stress, sweets consumption, inadequate face wash, and makeup are acne exacerbating factors, whilst cosmetic treatment, increased water consumption, smoking, and being on school holidays are acne ameliorating factors. Boys more frequently considered the benefit of losing weight. There was no statistically significant difference between pupils with and without acne in perceived factors, except for cosmetic treatment. Those with acne more frequently believed in the benefits of cosmetic treatment.

**Conclusions:**

Many adolescents’ self-perceived beliefs about factors that aggravate and ameliorate acne are myths and misconceptions without evidence-based justification. More efforts are needed to educate pupils about the acne aggravating and ameliorating factors, its health-related consequences, and the treatment possibilities.

## Introduction

Acne vulgaris is a common chronic inflammatory skin disease. A growing body of literature supports the high prevalence of acne worldwide, particularly in young people. It is estimated that up to 90% of adolescents are affected by acne [1, 2]. In addition to the physical impact, acne can have an emotional and psychological impact on sufferers regardless of its severity [3, 4]. It is worth noting that this impact is aggravated by the sociological evaluation of today’s adolescents who are more concerned by their appearance in comparison to previous generations at the same age [5]. Despite acne being an almost universal condition in younger people, information about its epidemiology is lacking [6]. Multiple factors, such as hormonal, environmental, immunological, and genetic factors are thought to contribute to the development of acne [7]. It is of great importance for adolescents with acne to be conscious about the factors that aggravate or ameliorate acne, to seek medical help on time, and prevent severe clinical manifestations and their consequences, such as scarring and costly treatments. On the other hand, gaining a better understanding of adolescents’ beliefs about acne is also important for treatment strategy as well as for treatment compliance and adherence [8, 9]. The aim of this study was to assess the self-perceived beliefs of Montenegrin secondary school pupils about factors that aggravate and ameliorate acne and to identify misconceptions between these beliefs and available evidence-based medical facts. The second aim was to analyze potential sex-based differences in acne-related beliefs, as well as differences between pupils with and without acne.

In the recently published paper, we investigated the quality of life (QoL) of Montenegrin pupils with acne in the same population cohort and confirmed the negative impact of acne on their QoL [10].

To the best of our knowledge, this is one of a few studies on a representative sample in the Western Balkan region focused on adolescents’ perceived acne-related beliefs.

## Methods

### Study design and participants

This cross-sectional study of Montenegrin pupils was conducted in four randomly selected public four-year secondary schools out of six in Podgorica, the capital of Montenegro (population about 200,000 inhabitants). Because of the COVID-19 pandemic, only first and second-grade pupils who attended school were included in the study. The sample size (530) was calculated using the assumption of 95% Confidence Interval (CI), and a marginal error of 5%, enlarged in view of consideration of the loss of response. Researchers first obtained approval from the principal of each selected school to conduct the study during school hours. None of the selected schools refused to participate in the survey. A letter with a detailed explanation of the planned survey and its purposes was sent to the parents of the pupils a week before the distribution of the questionnaire to allow their children to participate in the study.

### Data collection

The data were collected during October and November 2020. On the day of the survey, the physician gave an additional explanation about acne and the research itself. Participation was voluntary and anonymous. All pupils were asked to fill in a self-administered questionnaire based on the literature data which included questions on age, sex, presence of acne, seeking medical help, perceived acne aggravating or triggering factors (15 questions), perceived acne ameliorating factors (8 questions), and sources of information about acne (7 questions). All questions, except questions on age and sex, were designed with two possible answers: “yes” and “no.” The response rate among pupils was 94%.

The study was approved by the Ethics Committee of the Faculty of Medicine at the University of Montenegro (No.: 2050/5; December 10, 2020). The written informed consent was obtained from pupils’ parents.

### Statistical analysis

Statistical analysis was performed with the Statistical Package for the Social Sciences, SPSS version 20.0 (SPSS Inc., Chicago, IL, USA). A two-tailed probability value of 0.05 was considered significant.

Categorical variables were presented as counts and percentages while continuous variable age was expressed as mean ± standard deviation. Binary logistic regression was used to model the association between gender (males/females) or acne presentation (no/yes) and potential exacerbating and ameliorating factors, as well as sources of information. Univariate analyses were first conducted, followed by multivariate analyses adjusted for gender or the presence of acne where appropriate. Results were presented as odds ratios (OR) with 95% confidence intervals (CI).

## Results

A total of 500 pupils were included in the current study, 234 (46.8%) boys and 266 (53.2%) girls. The mean age of pupils was 15.03 ± 0.50 (range 14−17), and the majority of them (80.4%) were 15 years old. Acne was self-reported in 249 (49.8%) pupils, whereas 251 (50.2%) did not report acne. Almost 80% of respondents with acne did not seek medical help.

Self-perceived factors which can cause or aggravate acne in surveyed pupils are presented in Tables 1 and 2.

**Table 1:** Self-perceived acne risk or aggravating factors among pupils according to sex.

Factor	All N (%) (N = 500)	Sex N (%)		Univariate logistic regression analysis			Multivariate logistic regression analysis*		
		Boys (234)	Girls (266)	OR	95% CI	*P*	OR*	95% CI	*P*
**Genetics (inherited)**	282 (56.4)	115 (49.1)	167 (62.8)	1.746	1.221–2.495	0.002	1.620	1.100–2.385	0.015
**Hormones**	420 (84.0)	185 (79.1)	235 (88.3)	2.008	1.231–3.275	0.005	1.486	0.870–2.540	0.147
**Stress**	339 (67.8)	132 (56.4)	207 (77.8)	2.711	1.840–3.996	0.000	1.889	1.227–2.906	0.004
**Greasy food**	363 (72.6)	166 (70.9)	197 (74.1)	1.170	0.789–1.733	0.435	0.750	0.470–1.195	0.226
**Sweets**	410 (82.0)	177 (75.6)	233 (87.6)	2.274	1.419–3.642	0.001	2.095	1.228–3.537	0.007
**Dairy**	155 (31.0)	64 (27.4)	91 (34.2)	1.381	0.942–2.026	0.098	1.210	0.789–1.853	0.382
**Sweating**	336 (67.2)	147 (62.8)	189 (71.1)	1.453	0.999–2.113	0.051	1.213	0.802–1.836	0.361
**Exercise**	89 (17.8)	37 (15.8)	52 (19.5)	1.294	0.814–2.057	0.276	1.147	0.682–1.930	0.606
**Sun exposure**	104 (20.8)	41 (17.5)	63 (23.7)	1.461	0.941–2.268	0.091	1.353	0.823–2.224	0.233
**Less sleep hours**	239 (47.8)	93 (39.7)	146 (54.9)	1.845	1.292–2.634	0.001	1.399	0.945–2.070	0.094
**Inadequate face wash**	425 (85.0)	186 (79.5)	239 (89.8)	2.284	1.373–3.800	0.001	1.768	1.009–3.097	0.047
**Smoking**	235 (47.0)	99 (42.3)	136 (51.1)	1.427	1.002–2.032	0.049	0.993	0.620–1.582	0.977
**Alcohol**	196 (39.2)	76 (32.5)	120 (45.1)	1.709	1.186–2.461	0.004	1.384	0.845–2.267	0.197
**Coffee**	138 (27.6)	54 (23.1)	84 (31.6)	1.538	1.032–2.293	0.034	0.974	0.603–1.574	0.914
**Cosmetics/makeup**	356 (71.2)	143 (61.1)	213 (80.1)	2.557	1.715–3.813	0.000	1.883	1.205–2.944	0.005

*Adjusted for the presence of acne; OR: odds ratio; CI: confidence intervals.

**Table 2:** Self-perceived acne risk or aggravating factors among pupils according to the presence of acne.

Factor	All N (%) (N = 500)	Acne presence N (%)		Univariate logistic regression analysis			Multivariate logistic regression analysis*		
		No (N = 251)	Yes (N = 249)	OR	95% CI	*P*	OR*	95% CI	*P*
**Genetics (inherited)**	282 (56.4)	139 (55.4)	143 (57.4)	1.087	0.763–1.548	0.644	1.097	0.760–1.583	0.622
**Hormones**	420 (84.0)	217 (86.5)	203 (81.5)	0.691	0.427–1.121	0.134	0.708	0.427–1.172	0.180
**Stress**	339 (67.8)	180 (71.7)	159 (63.9)	0.697	0.478–1.016	0.061	0.763	0.504–1.155	0.201
**Greasy food**	363 (72.6)	188 (74.9)	175 (70.3)	0.792	0.534–1.175	0.247	0.781	0.505–1.208	0.267
**Sweets**	410 (82.0)	205 (81.7)	205 (82.3)	1.045	0.662–1.650	0.849	1.126	0.680–1.865	0.645
**Dairy**	155 (31.0)	70 (27.9)	85 (34.1)	1.340	0.916–1.961	0.131	1.447	0.973–2.152	0.068
**Sweating**	336 (67.2)	168 (66.9)	168 (67.5)	1.025	0.705–1.489	0.898	1.044	0.705–1.544	0.831
**Exercise**	89 (17.8)	43 (17.1)	46 (18.5)	1.096	0.693–1.734	0.695	1.071	0.661–1.735	0.780
**Sun exposure**	104 (20.8)	56 (22.3)	48 (19.3)	0.832	0.539–1.282	0.404	0.833	0.526–1.318	0.434
**Less sleep hours**	239 (47.8)	124 (49.4)	115 (46.2)	0.879	0.619–1.249	0.471	0.911	0.627–1.323	0.623
**Inadequate face wash**	425 (85.0)	213 (84.9)	212 (85.1)	1.022	0.626–1.670	0.930	1.146	0.682–1.928	0.607
**Smoking**	235 (47.0)	126 (50.2)	109 (43.8)	0.772	0.543–1.098	0.150	0.785	0.505–1.221	0.283
**Alcohol**	196 (39.2)	104 (41.4)	92 (36.9)	0.828	0.578–1.187	0.304	0.962	0.605–1.528	0.869
**Coffee**	138 (27.6)	70 (27.9)	68 (27.3)	0.971	0.656–1.438	0.885	1.088	0.696–1.700	0.713
**Cosmetics/makeup**	356 (71.2)	185 (73.7)	171 (68.7)	0.782	0.531–1.153	0.215	0.847	0.553–1.296	0.444

*Adjusted for sex; OR: odds ratio; CI: confidence intervals.

The top three factors that aggravate acne, reported by pupils were inadequate face washing (85.0%), hormones (84.0%), and sweets consumption (82.0%). More than two-thirds of pupils believed that consumption of greasy food (72.6%), makeup (71.2%), stress (67.8%), and sweating (67.2%) aggravate acne. Girls more frequently than boys believed that genetics (OR = 1.62; 95% CI = 1.10–2.39), stress (OR = 1.89; 95% CI = 1.23–2.91), sweets consumption (OR = 2.09; 95% CI = 1.23–3.54), inadequate face wash (OR = 1.77; 95% CI = 1.01–3.10), and makeup (OR = 1.89; 95% CI = 1.20–2.94) can worsen acne (Table 1).

There was no statistically significant difference between pupils with and without acne in the perceived factors that aggravate acne (Table 2).

Self-perceived factors which ameliorate acne in surveyed pupils are presented in Tables 3 and 4.

**Table 3:** Self-perceived acne ameliorating factors among pupils according to sex.

Factor	All N (%) (N = 500)	Sex N (%)		Univariate logistic regression analysis			Multivariate logistic regression analysis*		
		Boys (234)	Girls (266)	OR	95% CI	*P*	OR*	95% CI	*P*
**Diet change**^†^	387 (77.4)	175 (74.8)	212 (79.7)	1.324	0.870–2.014	0.191	1.082	0.693–1.689	0.728
**Gaining weight**	20 (4.0)	13 (5.6)	7 (2.6)	0.459	0.180–1.172	0.103	0.402	0.139–1.165	0.093
**Losing weight**	144 (28.8)	75 (32.1)	69 (25.9)	0.743	0.504–1.095	0.133	0.636	0.419–0.964	0.033
**Water hydrate**	388 (77.6)	162 (69.2)	226 (85.0)	2.511	1.624–3.883	0.000	2.400	1.506–3.825	<0.0001
**Sun exposure**	151 (30.2)	63 (26.9)	88 (33.1)	1.342	0.913–1.973	0.135	1.242	0.816–1.890	0.312
**Smoking**	21 (4.2)	8 (3.4)	13 (4.9)	1.452	0.591–3.566	0.416	2.775	1.014–7.597	0.047
**School holidays**	311 (62.2)	129 (55.1)	182 (68.4)	1.764	1.224–2.540	0.002	1.558	1.051–2.309	0.027
**Cosmetic treatment**	402 (80.4)	176 (75.2)	226 (85.0)	1.862	1.189–2.916	0.007	1.753	1.090–2.820	0.021

*Adjusted for the presence of acne

^†^toward healthier food choices; OR: odds ratio; CI: confidence intervals.

**Table 4:** Self-perceived acne ameliorating factors among pupils according to the presence of acne.

Factor	All N (%) (N = 500)	Acne N (%)		Univariate logistic regression analysis			Multivariate logistic regression analysis*		
		No (N = 251)	Yes (N = 249)	OR	95% CI	*P*	OR*	95% CI	*P*
**Diet change**^†^	387 (77.4)	194 (77.3)	193 (77.5)	1.013	0.666–1.540	0.953	0.956	0.622–1.471	0.838
**Gaining weight**	20 (4.0)	9 (3.6)	11 (4.4)	1.243	0.506–3.053	0.636	1.443	0.558–3.729	0.449
**Losing weight**	144 (28.8)	77 (30.7)	67 (26.9)	0.832	0.564–1.226	0.352	0.814	0.543–1.220	0.319
**Water hydrate**	388 (77.6)	194 (77.3)	194 (77.9)	1.036	0.681–1.578	0.868	1.016	0.648–1.592	0.946
**Sun exposure**	151 (30.2)	73 (29.1)	78 (31.3)	1.112	0.759–1.630	0.585	1.168	0.779–1.749	0.453
**Smoking**	21 (4.2)	11 (4.4)	10 (4.0)	0.913	0.381–2.190	0.838	0.884	0.346–2.255	0.796
**School holidays**	311 (62.2)	156 (62.2)	155 (62.2)	1.004	0.700–1.442	0.982	0.988	0.673–1.451	0.953
**Cosmetic treatment**	402 (80.4)	193 (76.9)	209 (83.9)	1.570	1.003–2.457	0.048	1.600	1.009–2.537	0.045

*Adjusted for sex

^†^toward healthier food choices; OR: odds ratio; CI: confidence intervals.

Most pupils believed in the benefits of cosmetic treatment of acne (80.4%). The other most prevalent acne ameliorating factors were increased water consumption (77.6%), a diet change to a healthier food choices (77.4%), and being on school holidays (62.2%). Girls more frequently than boys reported increased water consumption (OR = 2.40; 95% CI = 1.51–3.82), smoking (OR = 2.78; 95% CI = 1.01–7.60), being on school holidays (OR = 1.56; 95% CI = 1.05–2.31), and cosmetic treatment (OR = 1.75; 95% CI = 1.09–2.82) as acne ameliorating factors, while boys more frequently believed in the benefit of losing weight (OR = 0.64; 95% CI = 0.42–0.96) (Table 3).

There was no statistically significant difference between pupils with and without acne in the perceived factors that ameliorate acne, except for cosmetic treatment. Pupils with acne were more frequently convinced of the beneficial effects of cosmetic treatment in comparison to pupils without acne (OR = 1.60; 95% CI = 1.01–2.54) (Table 4).

Adolescents often seek information about acne from a variety of sources. In the present study the most frequent source of information was the internet (70.2%), followed by parents (65.0%), and friends (42.2%) (Tables 5 and 6).

**Table 5:** Source of information about acne among pupils according to sex.

Source	All N (%) (N = 500)	Sex N (%)		Univariate logistic regression analysis			Multivariate logistic regression analysis*		
		Boys (234)	Girls (266)	OR	95% CI	*P*	OR*	95% CI	*P*
**Parents**	325 (65.0)	152 (65.0)	173 (65.0)	1.004	0.694–1.450	0.985	1.068	0.715–1.595	0.748
**Doctor**	121 (24.2)	54 (23.1)	67 (25.2)	1.122	0.744–1.693	0.582	1.154	0.719–1.850	0.553
**Pharmacist**	88 (17.6)	31 (13.2)	57 (21.4)	1.786	1.107–2.881	0.017	1.579	0.922–2.704	0.096
**Friends**	211 (42.2)	93 (39.7)	118 (44.4)	1.209	0.846–1.726	0.297	0.999	0.685–1.457	0.994
**Internet**	351 (70.2)	144 (61.5)	207 (77.8)	2.193	1.483–3.243	0.000	2.295	1.510–3.489	<0.0001
**TV**	148 (29.6)	64 (27.4)	84 (31.6)	1.226	0.833–1.805	0.302	0.829	0.537–1.278	0.395
**Magazines**	135 (27.0)	41 (17.5)	94 (35.3)	2.573	1.690–3.917	0.000	2.711	1.718–4.278	<0.0001

*Adjusted for the presence of acne; OR: odds ratio; CI: confidence intervals.

**Table 6:** Source of information about acne among pupils according to the presence of acne.

Source	All N (%) (N = 500)	Acne presence N (%)		Univariate logistic regression analysis			Multivariate logistic regression analysis*		
		No (N = 251)	Yes (N = 249)	OR	95% CI	*P*	OR*	95% CI	*P*
**Parents**	325 (65.0)	142 (56.6)	183 (73.5)	2.128	1.461–3.100	0.000	2.056	1.382–3.059	<0.0001
**Doctor**	121 (24.2)	54 (21.5)	67 (26.9)	1.343	0.890–2.026	0.160	0.766	0.478–1.228	0.269
**Pharmacist**	88 (17.6)	24 (9.6)	64 (25.7)	3.272	1.969–5.436	0.000	3.504	2.016–6.090	<0.0001
**Friends**	211 (42.2)	103 (41.0)	108 (43.4)	1.101	0.772–1.570	0.597	1.141	0.782–1.665	0.492
**Internet**	351 (70.2)	182 (72.5)	169 (67.9)	0.801	0.545–1.176	0.257	0.803	0.527–1.225	0.309
**TV**	148 (29.6)	83 (33.1)	65 (26.1)	0.715	0.486–1.052	0.089	0.835	0.544–1.282	0.409
**Magazines**	135 (27.0)	78 (31.1)	57 (22.9)	0.658	0.442–0.981	0.040	0.656	0.418–1.028	0.066

*Adjusted for sex; OR: odds ratio; CI: confidence intervals.

Girls more frequently than boys reported internet (OR = 2.29; 95% CI = 1.51–3.49), and magazines (OR = 2.71; 95% CI = 1.72–4.28) as information sources (Table 5). Parents (OR = 2.06; 95% CI = 1.38–3.06) and pharmacists (OR = 3.50; 95% CI = 2.02–6.09) were more frequently reported by pupils with acne compared to those without acne (Table 6).

## Discussion

The prevalence of self-reported acne in the present study was about 50%, supporting the estimation that acne is a very common skin disease in adolescents [8, 11−15].

In the past two decades, numerous studies that evaluated beliefs and perceptions about acne in adolescents found that knowledge about this condition was poor [8, 13−16]. According to the results of the French study, almost 90% of adolescents did not perceive acne as a disease, but rather as a normal phase of adolescence [8]. Myths and misconceptions about the factors that exacerbate and ameliorate acne still exist among adolescents [8, 15], even among physicians [17].

More than two-thirds of all respondents in the present study believed that inadequate face washing (85.0%), hormones (84.0%), dietary factors, such as sweets consumption (82.0%) and greasy food (72.6%), cosmetics/makeup (71.2), stress (67.8), and sweating (67.2%) are exacerbation factors of acne. Over one-half of pupils (56.4%) indicated genetics as aggravating factors, while lack of sleep and smoking were considered to be risk factors for almost a half of pupils (47.8% and 47.0%, respectively). Our findings are consistent with other studies which reported that poor diet [11, 15, 18–24], hormones/female menstrual cycle [1, 8, 11, 14, 15, 18−21, 23, 25, 26], inadequate skin hygiene [8, 11, 15, 18−21, 23], and genetic factors [8, 13, 18, 20, 21] are the most important factors contributing to acne. Commonly identified acne aggravating factors also include stress [8, 12, 14, 15, 22, 23, 25, 27–30], excessive sweating [8, 15], makeup use [8, 15], lack of sleep [15, 22], and smoking [8, 31].

In the present study, the majority of pupils, more frequently girls and those with acne, believed in the benefits of cosmetic treatment of acne that is consistent with a recently published Serbian study results [15]. According to Polish authors, cosmetic treatment significantly improved the overall QoL of patients with acne [32]. Similar to the Serbian study, other reported ameliorating factors in our study included increased water consumption, a diet change to a healthier food choices, and being out on school holidays [15].

One of the aims of the present study was to analyze sex differences in acne aggravating and ameliorating factors. Montenegrin girls compared to boys more frequently reported genetics, stress, sweets consumption, inadequate face wash, and makeup as acne exacerbating factors. Several studies reported sex-based differences in adolescents’ acne-related beliefs with different results [14, 15, 23]. In the Serbian study girls significantly more frequently believed that emotional stress, sweets consumption, fatty foods, sun, and lack of sleep aggravate acne, whereas boys significantly more frequently reported sweating, exercise, and dairy foods [15]. Stratification according to sex in the Greek study revealed that a statistically significant percentage of girls believed that diet was implicated in acne causality [23]. In the study from Saudi Arabia, gender was significantly related to the level of knowledge about acne, with poorer knowledge in male students [14].

Self-perceived acne-related beliefs of Montenegrin pupils were shown to be similar to those of the adolescents in other countries. However, when comparing the results of our study with the results of other similar studies, it is necessary to take into account the socio-demographic, and cultural differences of the respondents in different settings. Also, it is important to underline that only a few of the most frequently reported acne aggravating and ameliorating factors in the present study and other previously reported, mainly cross-sectional studies, are supported by the evidence-based literature.

In the last decade, several observational studies and a few systematic reviews and meta-analyses of relevant epidemiological studies on potential factors that exacerbate and ameliorate acne have been performed. Despite some controversial findings, diet, hormones, genetics, emotional stress, and lifestyle factors are thought to play roles.

The relationship between diet and acne has been controversial [33, 34]. Western diet, characterized by high glycaemic load and high dairy protein consumption, has been suggested to be an important nutritional factor promoting the acne epidemic [35]. A large systematic review and meta-analysis of 14 observational studies (n = 78,529; 23,046 acne-cases/55,483 controls aged 7–30 years) concluded that dairy consumption was associated with an increased OR for acne [36]. While several studies have shown that high chocolate intake was a significant risk factor for acne [2, 37], other studies did not find any association [38, 39]. The results of a recently published meta-analysis [34] pointed out that high chocolate intake may increase the risk of acne. However, this result should be interpreted with caution due to the use of loose meta-analysis criteria [34]. Tan and Bhate [33] suggested that the sugars in dairy products and chocolate trigger insulin secretion, activating signalling pathways that lead to increased keratinocyte proliferation, and consequently to the formation of acne lesions. The beneficial therapeutic effect of a low glycaemic load diet in acne patients was confirmed in two randomized controlled studies [40, 41]. Consumption of high-fat food may also increase the risk of acne presentation, although the evidence is insufficient [34].

High fish, fruits, and vegetable consumption are suggested to lower the risk of acne development [31]. High levels of omega-3 fatty acids found in fish and the high fiber content in fruits and vegetables may reduce acne risk by decreasing the Insulin-like growth factor 1 (IGF-1) level and increasing sex hormone-binding globulin (SHBG) level [42].

Hormonal factors have commonly been associated with acne presentation. However, no relationship was found between the use of oral contraceptives or menstrual cycle patterns and acne [38, 34]. Karciauskiene et al. [26] found that acne in pubertal girls was three times, and in pubertal boys almost five times higher, compared with pre-pubertal schoolchildren.

Several studies have pointed out that family history of acne increases acne risk [1, 6, 34, 38, 43]. A cross-sectional population-based online survey of adolescents in Belgium, Czech and Slovak Republics, France, Italy, Poland, and Spain, reported that a history of maternal or paternal acne was associated with an increased probability of having acne (odds ratio 3.18, 95% CI 2.74 to 3.45, and 2.70, 95% CI 2.39 to 3.05, respectively) [1]. Bhate and Williams [6] reported that the heritability of acne was almost 80% in the first-degree relatives. A large twin study of acne in women provided supporting evidence that genetic factors play an important role in determining susceptibility to acne [44]. A recently published genetic study suggests that the MMP2 (−1306 C/T) polymorphism, in combination with the TIMP2 (−418 G/C) polymorphism, is associated with an increased risk of acne [7]. However, the effects of these polymorphisms on MMP2 gene activity and the risk of acne should be further explored.

A recently published meta-analysis [34] confirmed the finding of previous studies [38, 45] that overweight/obese body mass index (BMI) significantly influences acne presentation. Higher BMI may be associated with higher glycemic load and higher androgen levels, which may increase sebum production, promoting the formation of acne lesions [26]. However, dietary factors may confound the relationship between BMI and acne presentation.

There is substantial evidence of the association between emotional stress and acne presentation [46]. The mechanisms of stress-induced triggering or aggravation of acne involve the hypothalamus-pituitary-adrenal axis and the neuro-immuno-cutaneous system where neuropeptides and hormones such as corticotropin-releasing hormone, melanocortins, and substance P-containing nerves and mast cells, play an important role [47–50]. However, these mechanisms have not yet been completely understood.

The effect of smoking status on acne prevalence is controversial. While some observational studies found a positive relationship between smoking and acne prevalence [51–53], others found a negative association [1, 54, 55], or no association [38, 56, 57].

As already stated, diet, hormones, genetics, and emotional stress were recognized as factors related to acne by more than half of our study participants.

In the present study the most frequent source of information about acne was the internet (70.2%), followed by parents (65.0%), and friends (42.2%). Information about acne in two similar studies was obtained primarily from family physicians, mass media, and friends [17, 21]. More than a quarter (26.7%) of Greek high school acne patients received information about acne from a specialist dermatologist and only 1.1% from other doctors [23]. In a recently published study in adolescents and young adults with acne, dermatologists, and internet/social media were the most frequent sources of information [16]. Regarding seeking medical advice, only 20% of pupils with acne in the present study visited a physician, which is in accordance with several other studies [8, 13].

To our knowledge, this is the first study on adolescents’ perceptions of acne-related beliefs in Montenegro and one of the few studies in the Western Balkans region. The strengths of our study are a representative sample of secondary school pupils from Podgorica, the inclusion of both pupils with and without acne, as well as a high participation rate.

However, it is not possible to generalize our research findings to all secondary school pupils in Montenegro. Other limitations of the study include self-reported data on acne presence and the narrow age range (14−17 years) of pupils.

Nevertheless, this study showed that knowledge about the factors that aggravate and ameliorate acne is insufficient among Montenegrin pupils regardless of the presence of acne. The majority of them, more frequently girls than boys, believed that inadequate face washing, hormones, and sweets consumption worsen acne, whereas cosmetic treatment and increased water consumption improve acne. The low percentage of pupils with acne who visited physicians (20%) is the reflection of poor knowledge and misconceptions about the disease. More efforts are needed to educate pupils about acne aggravating and ameliorating factors, its health-related consequences, and the possibilities of effective treatment and control.

## Supporting information

S1 File Dataset. (XLS)
